# Epidemiology of Bovine Trypanosomiasis in Abe Dongoro District, Western Ethiopia: A Cross‐Sectional Study

**DOI:** 10.1155/japr/6290672

**Published:** 2026-05-23

**Authors:** Abdu Muhammed, Mitiku Wamile, Negasa Tamasgen, Belay Beyene, Megarsa Gemechu

**Affiliations:** ^1^ Department of Animal Science, Wollega University, Shambu, Ethiopia, wollegauniversity.edu.et

**Keywords:** Abe Dongoro district, bovine, prevalence, risk factors trypanosomosis

## Abstract

**Background and Aims:**

Bovine trypanosomosis is a major protozoan disease constraining cattle productivity in sub‐Saharan Africa. This cross‐sectional study is aimed at determining the prevalence and associated risk factors of bovine trypanosomosis in the Abe Dongoro district, Western Ethiopia.

**Methods:**

A cross‐sectional study was conducted between June 2023 and October 2023. A total of 520 randomly selected indigenous cattle were examined using buffy coat and Giemsa‐stain thin smear techniques for parasitological and hematological analysis. Packed cell volume (PCV) was measured to assess anemia. Data were analyzed using chi‐square tests, independent *t*‐tests, and multivariate logistic regression.

**Results:**

The overall prevalence of bovine trypanosomes was 20.96% (109/520; 95% CI:17.6–24.7). *T. congolense* was the predominant species (39.45%; 95% CI: 30.8–48.7), followed by *T. vivax* (24.77%; 95% CI: 17.8–33.3), *T. brucei* (22.02%; 95% CI: 15.1–30.8), and mixed infections (13.6%; 95% CI: 8.3–21.6). The prevalence was significantly higher in cattle with a poor body condition (AOR = 3.12; 95% CI: 1.82–5.34; *p* < 0.001), of adult age (AOR = 1.87; 95% CI: 1.06–3.29; *p* = 0.041), and with a black coat color (AOR = 8.94; 95% CI: 4.78–16.72; *p* < 0.001). Anemia (PCV < 24) was present in 21.54% (112/520) of cattle, with parasitemic animals showing significantly lower mean PCV (mean difference = 6.5 percentage points; *t* = −20.55; *p* < 0.001).

**Conclusion:**

Bovine trypanosomiasis remains a major constraint to cattle health and productivity in the Abe Dongoro district, highlighting the need for sustained vector control, routine screening, and integrated disease management strategies.

## 1. Introduction

Bovine trypanosomiasis is a hemoparasitic disease caused by unicellular protozoan parasites of the genus *Trypanosoma*, which inhabit the blood and various tissues of vertebrate hosts including livestock, wildlife, and humans [1, 2]. African animal trypanosomosis (AAT) primarily occurs in areas of Africa that are inhabited by its biological vector, the tsetse fly [3]. In Africa, where there is the greatest potential for substantial increases in domestic livestock productivity, trypanosomosis is one of the major health factors contributing to poor or negligible livestock output due to animal health constraints [4]. Approximately 50 million head of cattle and other livestock species are at risk due to the disease′s widespread distribution [5].

In most of African countries, particularly in sub‐Saharan Africa, African trypanosomiasis (commonly known as nagana) is a major livestock disease and remains a significant constraint to animal health and productivity [6]. *Trypanosoma vivax*, *Trypanosoma congolense*, and *Trypanosoma brucei* species are the culprits behind this disease. The interaction of the parasite, host, and environmental variables determines the course of disease incidence [7]. Several studies have reported that the prevalence of bovine trypanosomiasis in Africa varies based on the tsetse belt [8–10]. The disease′s spread and incidence in the afflicted region, together with the presence of its vectors, greatly influence its epidemiology and effects on livestock, particularly cow productivity [11]. They are a significant source of foreign exchange earnings for some nations and account for a sizable amount of the continent′s gross domestic product. Livestock play a crucial role in agricultural production, serving both as a direct source of food and as a provider of draught power for farming activities [12]. Ethiopia is one of the African nations with the greatest livestock population, and a significant part of the agricultural sector is not mechanized [13].

Although Ethiopia has a huge livestock population, it does not make the best use of this resource because of a number of issues, including inadequate nutrition, management issues with reproductive insufficiency, and animal disease [4]. Trypanosoma protozoan parasites are the primary cause of this hemoparasitic disease in domestic animals. The presence of appropriate tsetse habitat is a key factor in determining the prevalence and spread of bovine trypanosomosis [14]. Tsetse flies (*Glossina*) are found in 37 nations, including Ethiopia. Their diverse habitats span over 10 million km^2^, or 37% of the African continent, and 50 million people are exposed to human sleeping sickness. Additionally, roughly 30% of all cattle on the African continent are exposed to animal trypanosomosis [11]. One of the primary obstacles to livestock production and the full utilization of land to feed the world′s rapidly growing population is animal trypanosomosis, or Ghendi in Ethiopia. With the exception of the highlands, this illness is widespread in Ethiopia and is spread via mechanical or cyclical vectors. The majority of this disease is spread mechanically by biting flies, including *Stomoxys* and tabanids. Trypanosomosis is cyclically spread by *Glossina* (tsetse flies), which infect 180,000–200,000 km^2^ of productive agricultural area in the west and southwest regions of the nation [14]. In Ethiopia, bovine trypanosomiasis is one of the major constraints to livestock and a key barrier to optimal utilization of agricultural land for sustaining the world′s rapidly growing population [15].

There are five species of *Glossina* that are often found in Ethiopia: *Glossina morsitans submorsitans*, *G. fuscipes*, *G. tachnoids*, *G. pallidipes*, and *G. longipennis* [16]. *T. congolense* and *T. brucei* are the cyclically transmitted trypanosome species that infect cattle, sheep, and goats [17]. Trypanosomosis is cyclically transmitted in cattle and is a major problem in the country′s west and southwest agriculturally productive regions [18, 19]. The amount of interaction, the presence of infected cattle, the insect reservoir, and the seasons will all affect the percentage of trypanosomiasis [20]. There are a number of ways to diagnose this disease, including wet mount, buffy coat analysis, and the polymerase chain reaction (PCR) technique, which has a higher sensitivity than traditional parasitological procedures. PCR applications produce accurate amplification of products from the specific disease agent of interest, which can guide treatment choices to be carried out as soon as it is feasible in the field that supports better control programs [18, 19]. Trypanosomiasis in cattle has been managed using several preventative measures in areas where the disease is present. The most effective trypanosomosis preventive strategy nowadays mostly relies on controlling the disease vector, which involves setting up tsetse‐fly traps and applying pesticides to tsetse vectors [21]. The creation of a vaccine for the disease is difficult because the causal agent may evade the animals′ defense mechanisms in a variety of ways that combine immune suppression with antigenic polymorphism.

To maintain production in areas with a high fly burden, trypanotolerance in cattle breeds is another efficient way to prevent infectious diseases. Trypanotolerant West African taurine dairy cattle are likely to be a viable biological option for preventing clinical trypanosomosis and, consequently, lowering financial losses for the animal owners. Short‐horned and long‐horned *Bos taurus* breeds from West Africa, including the N′Dama and Baoule breeds, exhibit trypanotolerance [22]. Isometamidium chloride, diminazene aceturate, and homidium are the main medications currently used to treat AAT in trypanosome‐endemic areas; however, the emergence of drug resistance to the illness poses a significant challenge to disease control [23]. Despite numerous studies on bovine trypanosomosis in Ethiopia [18, 19], critical knowledge gaps remain for the Abe Dongoro district in the Horo Guduru Wollega zone. Specifically, no baseline data exist on the prevalence, risk factors, or spatial distribution of trypanosomosis in this district. Recent population movements (including resettlement programs) coupled with climate change have likely expanded tsetse habitats, yet the epidemiological consequences remain unquantified. The unresolved question of which local risk factors (body condition, age, coat color, or peasant association [PAs]) drive infection prevalence in this understudied area limits evidence‐based intervention design. Therefore, the objective of this study was to determine the prevalence and associated risk factors of bovine trypanosomosis in Abe Dongoro district, Oromia Regional State, and Western Ethiopia.

## 2. Materials and Methods

### 2.1. Study Area

**Figure 1:**
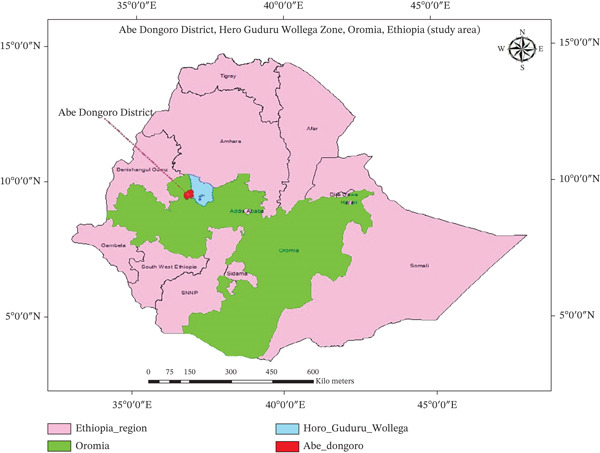
Map showing the study area (ArcGIS 2025).

The study was conducted in Abe Dongoro district, located in the western part of Horo Guduru Wollega zone, Oromia Regional State. Figure 1 shows a map of Ethiopia with the study area indicated. The district comprises 22 PAs and two urban PAs. It is located approximately 362 km northwest of Addis Ababa, which is the capital city of Ethiopia, and 47 km northwest of Shambu town. The district shares boundaries with Angar Gute (Gida Ayana district) to the west, Horo District to the east, Jardega Jarte district to the north, and Gudaya Bila district to the south. The district is situated at an altitude ranging from 1600 to 2300 m above sea level, with an average temperature between 12°C and 32°C, and an annual rainfall ranging from ,750 to 2750 mm; the livestock population of the district includes cattle (92,080), sheep (8292), goats (14,196), equine (9843), poultry (67,589), and the livestock are on a free‐grazing system [24].

### 2.2. Study Design

A cross‐sectional study was conducted to determine the prevalence of bovine trypanosomiasis and its associated risk factors in seven purposively selected PAs of Abe Dongoro district; Horo Guduru Wollega zone, from June 2023 to October 2023. The study is reported according to the STROBE‐Vet (Strengthening the Reporting of Observational Studies in Epidemiology Veterinary Extension) guidelines for cross‐sectional studies [25]. Local cattle of different age groups, different body condition scores, various colors, and both sexes were included from the selected study areas.

### 2.3. Study Animals

The study′s populations consisted of both sexes and local breeds of cattle reared under extensive production system, with communal grazing land, varying in age, body condition, and hair coat color. To determine whether an animal′s hair coat color has any bearing on the prevalence rate of a disease, the evaluated animals were divided into four groups based on their hair coat color: black, red, white, and gray, as recommended by [13]. Hair coat color was assessed visually by a trained veterinarian under natural daylight conditions. Interobserver agroecology was high (kappa = 0.89). Based on the dentition, cattle under examination were divided into three age groups: young (≤ 2 years), medium (2 to ˂ 4 years), and adult (≥ 4 years) [26]. The body condition of the cattle was estimated as recommended by [27]. Body condition was scored as good, medium, or poor based on rib and vertebral spine prominence [28]. Body condition scoring was performed independently by two trained assessors. Interobserver agroecology was assessed using weighted kappa (*κ* = 0.85, indicating substantial agroecology). Any disagro‐ecologies were resolved by consensus with a third assessor.

### 2.4. Sampling Methods and Sample Size Determination

A three‐stage sampling framework was employed for this study. In Stage 1 (purposive selection of PAs), seven PAs were purposively selected from the 22 PAs in the Abe Dongoro district based on accessibility and availability of transportation, logistical feasibility, and representation of different agroecological zones (highland, midland, and lowland). In Stage 2 (random selection of households), within each selected PA, households were selected using simple random sampling from the PA registry. In Stage 3 (random selection of cattle), within each selected household, individual cattle were selected using simple random sampling. This multistage approach ensures representativeness of the district′s cattle population (estimated at 92,080 heads), with a sample size (*n* = 520) providing adequate representation across the seven selected PAs. The required sample size (*n*) was determined according to the following formula described by [29], assuming an expected prevalence (p) of 50%, a 95% confidence level, and 5% desired absolute precision (d).

N=Z2x Pexp1−Pexpd2

where *n* is the required sample size, *Z* is equal to 1.96 (95% confidence level), Pexp is the expected prevalence, and *d* is the desired margin of error (precision).

A 50% expected prevalence was used to maximize sample size and ensure adequate power, as no prior study had been conducted in this specific district. Accordingly, based on the above formula, the minimum sample size required for the study would have been 384 cattle; however, 520 cattle were involved as the sample was increasing the precision of the study area. Increasing the sample size to 520 improved the precision of prevalence estimates (reducing the margin of error from 5% to approximately 4.3%) and allowed for stable subgroup analyses (across seven PAs and four coat color categories). Accordingly, based on population size, the PAs involved during the study periods were Gallesa (87), Charu (93), Garero (92), Gorte (79), Garero (92), Gorte (79), Botoro Bora (102), Tullu Wayu (40), and Tulu Moti (27).

### 2.5. Parasitological Examination and Sample Collection

#### 2.5.1. Buff Coat Techniques and PCV Measurement

A blood sample was obtained by puncturing the ear vein with a lancet and then placed in heparinized microhaematocrit tubes. Samples were taken to the veterinary laboratory within 30 min. The buffy coat technique was employed for diagnosis of trypanosome species, and species identification was based on the characteristics of motility observed in wet film preparations as previously described by [30]. After filling the tube to at least three‐fourths of its capacity, one end was sealed with a crystal seal [31]. The capillary tube′s end was sealed and centrifuged for 5 min at 12,000 rpm in order to separate the blood cells and extract trypanosomes. A hematocrit reader was then used to assess the packed cell volume (PCV) of red blood cells. The packed red blood cell column′s length is expressed as a proportion of the blood′s total volume. For negative samples, 100 oil immersion fields (100×) were examined before designating a slide as negative. Animals were classified as anemic if their PCV was less than 24% [32]. After breaking the capillary tubes about 1 mm below the buffy coat, they were placed on a microscopic slide, combined, and covered with a 22 × 22‐mm cover slip. In order to determine the trypanosome species based on their motility, the preparation was examined using a 40× objective of a microscopy using the dark ground buffy coat method [33]. Motility was used for initial positive/negative determination and parasitemia was not quantitatively measured; only presence or absence was recorded.

#### 2.5.2. Thin Blood Smear

A positive sample during buffy coat techniques was recorded, and thin blood films stained with Giemsa were used to identify the trypanosome species. After applying a tiny drop of blood from a microhaematocrit capillary tube to a clean slide and spreading it with another clean slide at a 45° angle, the slide was allowed to air‐dry in an upright position and fixed in methyl alcohol for 2 min. It was then submerged in Giemsa stain (1:10 solution) for 50 min. The smear was examined under a microscope using an oil immersion objective lens (100×) after removing excess stain with distilled water and allowing it to air‐dry in an upright position. Of the parasitological assays, this method is the most sensitive for identifying *T. vivax* and *T. congolense* [33, 34].

Confirmatory criteria for species identification of trypanosomes included: (i) for *T. congolense* (small size, kinetoplast marginal/subterminal, no free flagellum); (ii) for *T. vivax* (medium size, terminal kinetoplast, short free flagellum); (iii) for *T. brucei* (large size, subterminal kinetoplast, long free flagellum with prominent undulating membrane). In cases of uncertainty (*n* = 12), thin smears were re‐examined by a second experienced parasitologist blinded to the initial reading. Species identification followed OIE guidelines [30].

### 2.6. Statistical Analysis

All statistical analyses were performed using R Version 4.3.3 (R Core Team 2024). The a priori level of significance was set at *α* = 0.05, and all tests were two‐tailed. Prespecified analyses included prevalence by body condition, age category, sex, hair coat color, and PCV status. Exploratory analyses included prevalence variation among PAs. The following statistical tests were used: chi‐square (*χ* ^2^) tests for association between trypanosome infection and categorical risk factors (body condition, age, sex, coat color, and PA), with Fisher′s exact test used where small cell counts occurred; an independent two‐sample *t*‐test was used for comparing mean PCV values between parasitemic and aparasitemic groups and multivariate logistic regression was used to estimate adjusted odds ratios (AOR) and 95% confidence intervals (CI) for each risk factor while controlling for potential confounders (age, sex, body condition, coat color, and PA) [35]. Before logistic regression, multicollinearity among independent variables was assessed using the variance inflation factor (VIF), and all VIF values were below 2.5, indicating no significant multicollinearity. The Hosmer–Lemeshow goodness‐of‐fit test was used to assess model calibration (*χ* ^2^ = 8.24, *p* = 0.41), and model discrimination was evaluated using the area under the receiver operating characteristic curve (AUC = 0.79; 95% CI: 0.75–0.83). *p* Values were reported according to standard conventions: *p* < 0.001 for values less than 0.001; values between 0.001 and 0.01 were reported to three decimal places; values ≥ 0.01 were reported to two decimal places. For all analyses, *p* < 0.05 was considered statistically significant. Statistical terms and abbreviations are defined as follows: CI, confidence interval; OR, odds ratio; PCV, packed cell volume; SD, standard deviation; *χ* ^2^, chi‐square; t, t‐statistic; %, percentage; No, number [35].

### 2.7. Ethical Approval and Informed Consent

Ethical approval was obtained from the Wollega University Shambu Campus Institutional Review Board. Informed verbal consent was obtained from all cattle owners prior to sample collection, as approved by the IRB. All procedures involving animals were conducted in accordance with the university′s guidelines for animal welfare and research ethics.

## 3. Results

### 3.1. Prevalence of Trypanosomosis by PA

A total of 520 local cattle were randomly selected and examined, of which 109 tested positive for trypanosomosis, for an overall prevalence of 20.96% (109/520; 95% CI: 17.6 24.7). The prevalence of trypanosomosis was found among the seven PAs in Cheru (22.6%), Galesa (18.4%), Garero (20.6%), Gorte (19.0%), Botoro Bora (28.4%), Tulu Wayu (15.0%), and Tulu Moti (11.1%). The highest prevalence was recorded in Botoro Bora (28.4%; 95% CI: 20.4–37.9), whereas the lowest was observed in Tulu Moti (11.1%; 95% CI: 3.1–28.1). Spatial clustering was evident, with Botoro Bora PA showing notably higher prevalence (28.4%) than the district average (20.96%), suggesting localized transmission hotspots. Nevertheless, the differences in prevalence among the PAs were not statistically significant (*χ* ^2^ = 6.56, *p* = 0.37, as shown in Table 1).

**Table 1:** Prevalence by peasant association.

PA	No‐examined	No‐positive	Prevalence (%)	95% CI	*X* ^2^	*p* value
Charu	93	21	22.6	15.1–32.1		
Galesa	87	16	18.4	11.4–28.0		
Garero	92	19	20.6	13.4–30.3	6.56	0.37
Gorte	79	15	19.0	11.7–29.1		
Botoro Bora	102	29	28.4	20.4–37.9		
Tulu Wayu	40	6	15	6.8–29.4		
Tulu Moti	27	3	11.1	3.1–28.1		
**Total**	**520**	**109**	**20.96**	**17.6–24.7**		

### 3.2. Prevalence of Trypanosomosis and Its Association With Body Condition, Age, Sex, and Hair Coat Color

Among the indigenous cattle that tested positive for trypanosomosis, 19 (13.87%), 39 (17.1%), and 51 (32.9%) were observed to have good, medium, and poor body condition scores, respectively. There were statistically significant differences observed among body condition categories (*χ* ^2^ = 19.55, *p* < 0.001), as shown in Table 2. Similarly, data analysis among age categories revealed that there were statistically significant differences in prevalence (*χ* ^2^ = 6.92, *p* = 0.041). Of the 520 cattle examines (312 male and 208 female), the prevalence of trypanosomesis was higher in male (73, 23.39%) as compared with female (36, 17.3%). However, there was no statistically significant difference between the sex groups (*χ* ^2^ = 2.44, *p* = 0.095) as shown in (Table 2). Across the hair coat color of the cattle, the highest prevalence of trypanosomosis was recorded in black hair coat color (42.2%; 95% CI: 35.6–49.0), whereas the lowest prevalence was recorded in gray hair coat color (6.3%; 95% CI: 3.3–11.7), showing statistical significance among the hair coat color (*χ* ^2^ = 76.58, *p* < 0.001), as showed in Table 2.

**Table 2:** Prevalence by risk factors.

Risk factors	No‐examined	No‐positive	Prevalence (%)	95% CI	*X* ^2^	*p* value
**Body condition**						
Good	137	19	13.87	8.9–20.6		
Medium	228	39	17.1	12.7–22.6	19.548	0.001∗∗
Poor	155	51	32.9	25.8–40.8		

**Age category**						
Young	112	21	18.75	12.6–27.0		
Medium	238	41	17.23	12.9–22.5	6.9205	0.041∗
Adult	170	47	27.64	21.3–34.9		
**Sex**						
Male	312	73	23.39	18.9–28.5	2.438	0.095
Female	208	36	17.3	12.7–23.0		

**Hair coat color**						
Black	204	86	42.2	35.6–49.0		
White	80	6	7.5	3.4–15.5	76.58	0.0001∗∗
Red	93	12	13	7.5–21.3		
Gray	143	9	6.3	3.3–11.7		

*Note:* Double asterisks “∗∗” denote highly significant. Single asterisk “∗” denotes significant.

### 3.3. Prevalence of Trypanosome Species Among Infected Cattle

**Figure 2:**
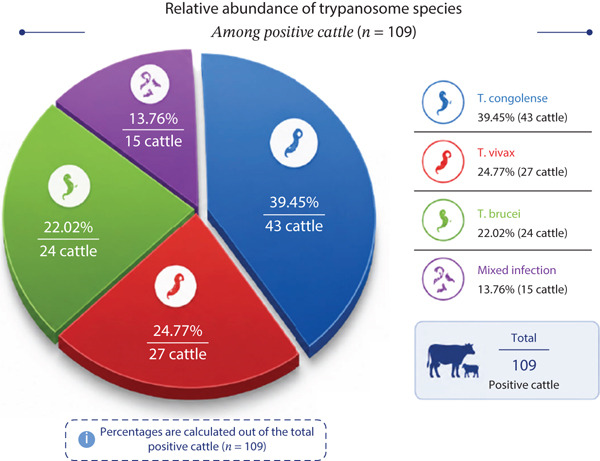
Trypanosome species distribution among positive cattle (*n* = 109).

Among 109 infected cattle examined, the most predominant trypanosome species detected were *T. congolense* 43 (39.45%; 95% CI: 30.8–48.7) followed by *T. vivax* 27 (24.77%; 95% CI: 17.8–33.3), *T. brucei 24 (3.22.02%;* 95% CI: 15.1–30.8), and mixed infection 15 (13.76%; 95% CI: 8.3–21.6), as shown in Figure 2. There was no statistically significant difference among the trypanosome species distribution (*χ* ^2^ = 18.15, *p* = 0.80), as shown in the supporting material.

### 3.4. Anemia and Parasitemia Association

Of the total number of cattle examined, 112 (21.54%) and 408 (78.46%) were anemic (PCV < 24) and nonanemic (PCV ≥ 24), respectively. Among the anemic cattle, 64 (58.72%) and 48 (11.68%) were found to be parasitemic and aparasitemic, respectively, as depicted in Table 3. In contrast, among aparasitemic and parasitemic animals, 363 (88.32%) and 45 (41.28%) were nonanemic (PCV ≥ 24), respectively. The strong anemia–parasitemia interaction is evident: parasitemic cattle were 5.2 times more likely to be anemic than aparasitemic cattle. The mean PCV values between parasitemic (mean = 21.3*%*, SD = 2.8) and aparasitemic (mean = 27.8*%*, SD = 3.4) cattle showed a statistically significant difference (*t* = −20.55, df = 518, *p* < 0.001).

**Table 3:** PCV status by trypanosome infection in the study area.

Condition	No observation	PCV ˂ 24%	PCV ≥ 24%	Mean PCV (SD)	*t*‐test	*p* value
Negative	411	48 (11.68%)	363 (88.32%)	27.8 (3.4)	−20.55	0.001∗∗
Positive	109	64 (58.72%)	45 (41.28%)	21.3 (2.8)		
**Total**	**520**	**112 (21.54%)**	**408 (78.46%)**			

*Note:* Asterisk "∗" indicates that the result is highly statistically significant at *p* < 0.01.

Abbreviations: df, degrees of freedom; PCV, packed cell volume; SD, standard deviation.

### 3.5. Multivariate Logistic Regression

Multivariate logistic regression (Table 4) identified poor body condition (AOR = 3.12; 95% CI: 1.82–5.34; *p* < 0.001), adult age (AOR = 1.87; 95% CI: 1.06–3.29; *p* = 0.041), and black coat color (AOR = 8.94; 95% CI: 4.78–16.72; *p* < 0.001) as independent risk factors.

**Table 4:** Multivariate logistic regression analysis of risk factors for bovine trypanosomosis (*n* = 520).

Risk factor	Risk factors	AOR	95% CI	*p* value
Body condition	Good (ref)	1.00	—	—
	Medium	1.28	0.74–2.21	0.38
	Poor	3.12	1.82–5.34	< 0.001∗

Age	Young (ref)	1.00	—	—
	Medium	0.91	0.52–1.60	0.74
	Adult	1.87	1.06–3.29	0.041

Sex	Female (ref)	1.00	—	—
	Male	1.46	0.93–2.29	0.10

Coat color	Gray (ref)	1.00	—	—
	White	1.21	0.41–3.57	0.73
	Red	2.15	0.86–5.37	0.10
	Black	8.94	4.78–16.72	< 0.001∗

*Note:* Model diagnostics: Hosmer–Lemeshow goodness‐of‐fit *χ* ^2^ = 8.24, *p* = 0.41; AUC = 0.79 (95% CI: 0.75–0.83). Asterisk "∗" indicates that the result is highly statistically significant at *p* < 0.01.

Abbreviations: AOR, adjusted odds ratio; CI, confidence interval; ref, reference category.

## 4. Discussion

This study found a bovine trypanosomosis prevalence of 20.96% in Abe Dongoro district, with *T. congolense* as the predominant species (39.5%). Poor body condition (AOR = 3.12), adult age (AOR = 1.87), and black coat color (AOR = 8.94) were independent risk factors. Bovine trypanosomiasis is the major protozoan parasitic disease of cattle in Ethiopia, leading to substantial economic losses through decreasing productivity and mortality. Its distribution shows marked variation across different ecological and geographical regions. The overall prevalence of bovine trypanosomosis in the study area was 20.96% (109/520; 95% CI: 17.6–24.7). This prevalence is comparable with reports from other Ethiopian districts but should be interpreted cautiously because traditional parasitological methods (buffy coat and thin smear) have lower sensitivity than PCR. Fluctuating parasitemia in infected cattle can lead to false negative results, potentially underestimating true prevalence. Prevalence of the bovine trypanosomiasis requires control and prevention strategies by policymakers. However, direct comparisons should be made cautiously, as these studies varied in diagnostic methods (buffy coat vs. PCR vs. wet mount), sampling seasons (dry vs. wet), and ecological settings (tsetse belt vs. mechanical transmission zones).

The result of this current finding was almost in line with previous documented as 21.5% at Bambasi woreda, Western Ethiopia [7] and the current finding was lower than the previous reported 27.5% in selected district of Arbamich, SNNP Southern Ethiopia [36], However the prevalence current finding was higher than the previous studies, 3.49% in Jimma Arjo district, East Wollega zone [37], 3.44% in Jawi district North West Ethiopia [38], 13.36% in Jimma Zone [39], 10.63% in Yem special district [17], 8.7% in Kellem Wollega Western Ethiopia [18], 12.24% in Botor Tolay district of Jimma zone [14], 3.7% in Jimma Horro district kellem Wollega zone [40], and 6.5% in Jima Geneti district of the Horo Guduru Wollega zone [41]. These variations in prevalence rate reported by various researchers might be due to the difference in agroecologically, vectors infection rate, sampling season, animal susceptibility, and practice of trypanocidal drug use tsetse (vectors) control methods, which may have impact on epidemiological of the diseases [41–43].

According to the samples collected across the PA within the district, the highest prevalence was recorded in Botoro Bora (28.4%), whereas the lowest was observed in Tulu Moti (11.1%), and there was no statistically significant difference among the PAs. The prevalence documented in the present study was higher than the earlier reports of Sodo Zuriya districts, Woliata zone, Southern Ethiopia [44], in Jimma Horo district, Oromia regional state, Western Ethiopia [45] and in Nono distrioct west shewa zone, Ethiopia [13], which reported 10.94%, 7.6%, and 6.7%, respectively.

The current finding shows that the prevalence rate of bovine trypanosomes was higher in poor body condition 32.9%, followed by medium 17.1% and good body condition 13.87%. This association was statistically significant (*χ* ^2^ = 19.55, *p* < 0.001). The result of this finding is consistent with the study reported by [17, 44, 46], who reported that there was a significant difference in the prevalence of bovine trypanosomiasis among cattle categorized according to their body condition status from Yem special district, southern nation, nationalities and SNNPR of Ethiopia, Gidami district of Kellem Wollega zone of Oromia regional state of Ethiopia and Sodo Zuriya district Woliata zone Southern, Ethiopia, respectively. In contrast, a statistically insignificant effect of body condition on the prevalence of trypanosomiasis in cattle was reported from Kindo koysha woreda of Wolaita zone of Ethiopia [47]. These differences might be attributed to the disease itself, which causes progressive emaciation in infected animals [48, 49].

Based on the current finding of this study, the predominant infection was caused by *T*. *congolense* (39.45%; 95% CI: 30.8**-**48.7), follwed by *T*. *vivax* (24.77%; 95% CI: 17.8 33.3), *T. brucei (*22.02%; 95% CI: 15.1–30.8), and mixed infection with *T. congolence* with *T. vivax* (13.76%; 95% CI: 8.3–21.6). Across all PAs, *T. congolense* remained the predominant species, suggesting consistent vector‐mediated transmission rather than mechanical transmission alone. This higher prevalence rate of infection of *T*. *congolence* in the study area was agroecology with diffent scholars [4, 17, 18, 50, 51]. The prevalence of *T*. *congolese* may be attributed the presence of its biological vectors (*Glossina* species). The high proportion of *T. congolense* suggests cyclical transmission by tsetse flies rather than mechanical transmission alone. *T. vivax* and *T*. *brucei* are capable of invading tissue [4, 19, 27, 51]. The mixed infections were increasingly being documented in bovine population, particularly in tsetse‐endemic and mechanically transmitting regions. For instance a study in Dabo Hana district, southwest Oromia, Ethiopia found that among livestock with trypanosomosis, of bovine had mixed infections [52] and another investigation in Bambasi woreda (Western Ethiopia) reported mixed infection [7]. The occurrence of mixed infections might be due to diseases severity, diagnosis, and vectors control method, as coinfection may influence parasite load and detection, whereas effective treatment and control management must target all circulating species rather than single pathogen [53].

Among the 520 cattle examined (312 males and 208 females), the prevalence of infection was higher in males (23.39%) compared with females (17.3%). However, the difference between the sexes was not statistically significant (*χ* ^2^ = 2.44, *p* = 0.095). This finding was in agroecology with previous reports from Bambasi woreda, Western Ethiopia [7], Etang district of Gambella, Ethiopia [54], and selected woredas of Gambella regional state, south Western Ethiopia [55]. This might be due to greater exposure from worked related movement, stress, and management practices, predisposing them to infection compared with females [7].

The prevalence rate among the age groups was found to be higher in adults (≥ 4 years) at 27.64% (95% CI: 21.3–34.9), followed by young (1–2 years) at 18.75% (95% CI: 12.6–27.0) and medium (2 to ≤ 4 years) at 17.23% (95% CI: 12.9–22.5), with age being statistically significant (*χ* ^2^ = 6.92, *p* = 0.041). This finding was in line with the previously reported data from Mareka woreda of Dawuro zone, Southern Ethiopia [56]. However, the variance in infection rates might be due to the mature animals traveling across large distances for grazing, and crop harvesting in tsetse‐challenged areas. Furthermore, suckling calves remain at home and do not accompany their mothers to grazing areas until weaning [57]. Young animals are potentially protected against infection through the passive transfer of maternal antibodies [56]. This may contribute to the low trypanosome prevalence. Additionally, tsetse flies had a harder time feeding on young cattle that were as young as 2 years old. Young animals have a lower feeding rate because they move more defensively, which lowers their chance of getting trypanosomosis [58].

The present study revealed a higher prevalence of infection in cattle with black coat color (42.2%; 95% CI: 35.6–49.0), with statistically significant differences (*χ* ^2^ = 76.58, *p* < 0.001).

Multivariate analysis confirmed black coat color as the strongest independent risk factor (AOR = 8.94; 95% CI: 4.78–16.72), even after controlling for age, body condition, and PA. This is consistent with reports from Wolaita zone, Southern Ethiopia [59]; this is explained by tsetse flies being naturally attracted to black coloration; consequently, different studies show cattle with black coat color exhibited high prevalence of trypanosomiasis [60, 61].

Anemia is regarded as an important clinical sign and or diagnostic indicator of trypanosomosis, and it is closely associated with the reduced productivity of infected animals [62]. Cattle with PCV values < 24% were considered anemic [63]. In the present study, 21.54% (112/520) of the total animals examined were anemic (PCV < 24%), whereas 78.46% (408/520) were nonanemic (PCV ≥ 24%). Among 109 parasitemic animals, 58.72% (64/109) were anemic (PCV < 24%), whereas 41.28% (45/109) were nonanemic (PCV ≥ 24%). The strong association between parasitemia and anemia (58.72% of parasitemic cattle anemic vs. 11.68% of aparasitemic cattle anemic) reflects the multifactorial pathogenesis of trypanosome‐induced anemia. Proposed mechanisms include: (i) immune‐mediated hemolysis (erythrophagocytosis and complement activation), (ii) bone marrow suppression due to proinflammatory cytokines (TNF‐*α*, IFN‐*γ*), (iii) oxidative stress from parasite‐derived factors, and (iv) nutritional stress exacerbating erythropoietic failure [64]. The significant mean PCV difference (6.5 percentage points) underscores the clinical and production impact of trypanosomosis in this setting. A statistically significant difference was observed between PCV value of parasitaemic and aparasitaemic animals (*t* = −87.4, *p* < 0.001). The finding was agroecology previous reports from Dara district of Sidama zone, Southern Ethiopia [65], West Gojam zone, Northwest Ethiopia [32], Dale Wabera district of Kellem Wollega zone Western Ethiopia [66], Zabo Gazo woreda, Southern Ethiopia [67], Sodo Zuriya district Woliata zone, Western Ethiopia [44]; Benatsemay district, South Omo zone, Ethiopia [13]. Anemia was more prevalent among parasitemic cattle (57.1%) than aparasitemic cattle (11.0%). However, some parasitemic animals did not exhibit low PCV. This may be explained by several factors: (i) early stage of infection before anemia develops, (ii) partial trypanotolerance in some indigenous cattle breeds, (iii) low parasitemia levels insufficient to induce significant erythrocyte destruction, or (iv) nutritional factors supporting erythropoiesis. Conversely, some aparasitemic animals were anemic, which may be attributed to other causes including gastrointestinal parasitism, malnutrition, or concurrent infectious diseases.

### 4.1. Limitations of the Study

This study has several limitations. First, the cross‐sectional design prevents determination of causal relationships between risk factors and infection. Second, parasitological diagnosis (buffy coat and thin smear) has lower sensitivity compared with molecular methods (PCR). Buffy coat sensitivity ranges from 70% to 85% compared with PCR (near 100%), meaning true prevalence may be underestimated by 15%–30%, particularly for chronic infections with parasitemia below the microscopic detection threshold (approximately 10^4^ trypanosomes/mL). Future studies incorporating PCR would provide more accurate prevalence estimates. Third, vector density and species distribution were not assessed. Incorporating Glossina trapping and infection rate analysis would strengthen causal inference between transmission pressure and infection prevalence. This is a priority for future research in the district. Fourth, although trypanocidal drug use history was recorded, recall bias was a limitation: 23% of cattle owners could not provide precise treatment dates or drug names. Additionally, the study did not verify drug quality or authenticity, and the possibility of drug resistance was not assessed. Consequently, the true effect of prior trypanocidal treatment on parasitemia detection could not be fully accounted for in the analysis.

### 4.2. Future Direction

The primary findings that black coat color (AOR = 8.94–10.82), adult age (AOR = 1.87), and poor body condition (AOR = 3.12) are significant risk factors for bovine trypanosomosis raise important questions for future investigation. Regarding coat color, potential explanations include greater tsetse attraction to dark hosts [68, 69], genetic linkage between coat color and immune response genes, or behavioral correlates such as grazing patterns. Future studies should conduct controlled field experiments comparing tsetse landing rates on different coat colors, perform genome‐wide association studies to identify genetic markers linking coat color and trypanotolerance, and investigate whether coat color proxies other heritable traits. Regarding age‐related susceptibility, longer cumulative vector exposure, waning immunity, or higher mobility of adult cattle to grazing areas are possible explanations; longitudinal cohort studies following cattle from birth would help distinguish these factors. Regarding anemia, 42.9% of anemic cattle were aparasitemic, suggesting other causes including gastrointestinal nematodes, babesiosis, anaplasmosis, or nutritional deficiencies. Future studies should investigate coinfections and nutritional status concurrently with trypanosomosis screening.

## 5. Conclusion and Recommendations

Bovine trypanosomosis is highly prevalent in Abe Dongoro district (20.96%). *T. congolense* is the predominant species. Poor body condition, adult age, and black coat color are significant risk factors. Higher prevalence and statistically significant differences were observed in poor body condition (*p* < 0.001), old age (*p* = 0.041), and black hair coat color (*p* < 0.001). The diseases remain a constraint to livestock production in the study area. Therefore, the key recommendations include targeted vector control using deltamethrin‐impregnated traps along grazing routes and riverine habitats, integration of trypanosomosis surveillance into district livestock health information systems, capacity building for community animal health workers in rapid diagnostic testing, strategic trypanocidal treatment protocols (diminazene aceturate for acute cases and isometamidium for prophylaxis), and stakeholder engagement among the district agricultural office, veterinary services, and cattle owner cooperatives for coordinated implementation.

## Author Contributions

**Abdu Muhammed:** conceptualization, methodology, investigation, data curation, writing original draft, writing review and editing, project administration. **Mitiku Wamile:** methodology, investigation, validation, writing review and editing. **Negasa Tamasgen:** investigation, formal analysis, writing review, and editing. **Belay Beyene:** investigation, resources, writing – review and editing. **Megarsa Gemechu**: formal analysis, software, writing review and editing.

## Funding

No funding was received for this manuscript.

## Disclosure

All authors have read and approved the final version of the manuscript. Abdu Muhammed had full access to all of the data in this study and takes complete responsibility for the integrity of the data and the accuracy of the data analysis. Abdu Muhammed affirms that this manuscript is an honest, accurate, and transparent account of the study being reported; that no important aspects of the study have been omitted; and that any discrepancies from the study as planned have been explained.

## Conflicts of Interest

The authors declare no conflicts of interest.

## Data Availability

The authors confirm that the data supporting the findings of this study are available within the article and its supporting materials. Raw data are available from the corresponding author upon reasonable request.

## References

[1] Beaver P. C. , Helminths, Arthropods and Protozoa of Domesticated Animals, American Journal of Tropical Medicine and Hygiene. (1983) 32, no. 4, 10.4269/ajtmh.1983.32.906.

[2] Gordon H. M. L. , Veterinary Parasitology, Australian Veterinary Journal. (1968) 44, no. 9, 405–405, 10.1111/j.1751-0813.1968.tb09132.x, 2-s2.0-84980084756.

[3] Yigzaw B. , Asmare T. , and Derso S. , Prevalence of Bovine Trypanosomiasis and Its Vector Density in Sheka Zone, Anderacha Woreda, Online Journal of Animal and Feed Research. (2017) 7, no. 3, 51–57.

[4] Gebisa G. , Beriso K. , Bogale B. , Gizaw O. , and Chala D. , Bovine Trypanosomosis and Its Vectors in Three Selected Districts of Buno Bedele Zone of Oromia Region, Ethiopia, Veterinary Medicine International. (2020) 2020, no. 1, 1571947, 10.1155/2020/1571947. 32774830 PMC7397448

[5] Degneh E. , Shibeshi W. , Terefe G. , Asres K. , and Ashenafi H. , Bovine Trypanosomosis: Changes in Parasitemia and Packed Cell Volume in Dry and Wet Seasons at Gidami District, Oromia Regional State, Western Ethiopia, Acta Veterinaria Scandinavica. (2017) 59, no. 1, 10.1186/s13028-017-0327-7, 2-s2.0-85029233797, 28893322. PMC5594549 28893322

[6] Abutarbush S. M. , Veterinary Medicine — A Textbook of the Diseases of Cattle, Horses, Sheep, Pigs and Goats, 10th Edition, Canadian Veterinary Journal. (2010) 51, no. 5.

[7] Tikuye Yalew S. , Prevalence of Bovine Trypanosomosis and Its Associated Risk Factors in Bambasi woreda, Western Ethiopia, Journal of Dairy, Veterinary & Animal Research. (2017) 5, no. 2, 10.15406/jdvar.2017.05.00132.

[8] Kikway C. and Ngeiywa M. , Prevalence of Bovine Trypanosomiasis in Kilifi County, Kenya, Journal of Crops, Livestock and Pest Management. (2023) 1, no. 1, 35–41, 10.69897/joclipm.v1i1.46.

[9] Kizza D. , Ocaido M. , Mugisha A. , Azuba R. , Nalule S. , Onyuth H. , Musinguzi S. P. , Okwasiimire R. , and Waiswa C. , Prevalence and Risk Factors for Trypanosome Infection in Cattle From Communities Surrounding the Murchison Falls National Park, Uganda, Parasites and Vectors. (2021) 14, no. 1, 1–7, 10.1186/s13071-021-04987-w, 34620230. 34620230 PMC8499574

[10] Okello I. , Mafie E. , Eastwood G. , Nzalawahe J. , Mboera L. E. G. , and Onyoyo S. , Prevalence and Associated Risk Factors of African Animal Trypanosomiasis in Cattle in Lambwe, Kenya, Journal of Parasitology Research. (2022) 2022, no. 1, 5984376, 10.1155/2022/5984376, 35872666. 35872666 PMC9303511

[11] Bitew M. , Amedie Y. , Abebe A. , and Tolosa T. , Prevalence of Bovine Trypanosomosis in Selected Areas of Jabi Tehenan District, West Gojam of Amhara Regional State, Northwestern Ethiopia, African Journal of Agricultural Research. (2011) 6, no. 1, 140–144, 10.5897/AJAR10.426.

[12] Tora E. and Dana D. , Epidemiology and Economic Cost of Trypanosomosis Among SmallHolder Cattle Herders in Arba Minch and Zuria Districts, Gamo Zone, Ethiopia, Environmental Health Insights. (2024) 18, 11786302241274698, 10.1177/11786302241274698, 39192969. 39192969 PMC11348359

[13] Muktar Y. , Asmelash M. , and Mekonnen N. , Prevalence and Associated Risk Factors of Bovine Trypanosomosis in Benatsemay District, Southomo Zone Ethiopia, Livestock Research for Rural Development. (2016) 28, no. 12, 4–10.

[14] Bekuma F. , Prevalence of Bovine Trypanosomosis and Apparent Density of Tsetse Fly in Botor Tolay District, Jimma Zone, Ethiopia, Biomedical Journal of Scientific & Technical Research. (2019) 13, no. 3, 002401, 10.26717/bjstr.2019.13.002401.

[15] Tsolo A. , Kore K. , and Sheferaw D. , Bovine Trypanosomosis, Vector Distribution and Infection Rate in Three Districts of Gamo Zone, Southwestern Ethiopia, Parasite Epidemiology and Control. (2024) 26, e00374, 10.1016/j.parepi.2024.e00374, 39282215. 39282215 PMC11395718

[16] Mulat G. , Maru M. , Tarekegn Z. S. , and Dejene H. , A Systematic Review and Meta-Analysis on Prevalence of Bovine Trypanosomosis in East Africa, Parasite Epidemiology and Control. (2024) 26, e00371, 10.1016/j.parepi.2024.e00371, 39184304. 39184304 PMC11341968

[17] Bekele D. , Wang X. , Beshir A. , and Terefe E. , Prevalence of Bovine Trypanosomosis and Tsetse Fly Density in the Yem Special District: A Cross-Sectional Study, Frontiers in Veterinary Science. (2024) 11, 1460650, 10.3389/fvets.2024.1460650, 39764366. 39764366 PMC11700795

[18] Tsegaye D. , Terefe G. , Delema D. , and Tadesse A. , Bovine Trypanosomosis and Its Vectors: Prevalence and Control Operations in Kellem Wollega, Western Ethiopia, Ethiopian Veterinary Journal. (2021) 25, no. 2, 60–84, 10.4314/evj.v25i2.5.

[19] Tsegaye G. , Abebe B. , and Haile G. , Prevalence of Bovine Trypanosomosis and Its Associated Risk Factor in Hawa Galan District, Kelem Wollega Zone of Ethiopia, Veterinary Medicine International. (2021) 2021, 1–6, 10.1155/2021/4531586, 34950445. PMC8692041 34950445

[20] Tafese W. , Melaku A. , and Fentahun T. , Prevalence of Bovine Trypanosomosis and Its Vectors in Two Districts of East Wollega Zone, Ethiopia, Onderstepoort Journal of Veterinary Research. (2012) 79, no. 1, 1–4, 10.4102/ojvr.v79i1.385, 2-s2.0-84911947274. 23327311

[21] Schofield C. J. and Maudlin I. , Trypanosomiasis Control, International Journal for Parasitology. (2001) 31, no. 5–6, 615–620, 10.1016/S0020-7519(01)00162-X, 2-s2.0-0035344701. 11334951

[22] Akol G. W. , Authié E. , Pinder M. , Moloo S. K. , Roelants G. E. , and Murray M. , Susceptibility and Immune Responses of Zebu and Taurine Cattle of West Africa to Infection With *Trypanosoma congolense* Transmitted by *Glossina morsitans centralis* , Veterinary Immunology and Immunopathology. (1986) 11, no. 4, 361–373, 10.1016/0165-2427(86)90038-3, 2-s2.0-0022530526, 3716196. 3716196

[23] Dagnachew S. , Tsegaye B. , Awukew A. , Tilahun M. , Ashenafi H. , Rowan T. , Abebe G. , Barry D. J. , Terefe G. , and Goddeeris B. M. , Prevalence of Bovine Trypanosomosis and Assessment of Trypanocidal Drug Resistance in Tsetse Infested and Non-Tsetse Infested Areas of Northwest Ethiopia, Parasite Epidemiology and Control. (2017) 2, no. 2, 40–49, 10.1016/j.parepi.2017.02.002, 2-s2.0-85014454820, 29774280. 29774280 PMC5952666

[24] Wamile M. , Muhammed A. , Gemechu M. , Beyene B. , and Tamasgen N. , Prevalence and Associated Risk Factors of Trypanosomosis in Small Ruminants of Abe Dongoro District, Western Ethiopia, Parasite Epidemiology and Control. (2026) 33, e00496, 10.1016/j.parepi.2026.e00496, 41940266. 41940266 PMC13049284

[25] Sargeant J. M. and O′Connor A. M. , Issues of Reporting in Observational Studies in Veterinary Medicine, Preventive Veterinary Medicine. (2014) 113, no. 3, 323–330, 10.1016/j.prevetmed.2013.09.004, 2-s2.0-84893757853, 24139690. 24139690

[26] Severinghaus C. W. , Tooth Development and Wear as Criteria of Age in White-Tailed Deer, Journal of Wildlife Management. (1949) 13, no. 2, 195–216, 10.2307/3796089.

[27] Dagnachew S. , Mohammed S. , Dessie B. , Tilahun M. , Ayele A. , and Kefyalew H. , Bovine and Equine Trypanosomosis in Northwest Ethiopia: Prevalence, Density of Vectors and Control Measures, Parasite Epidemiology and Control. (2020) 11, e00170, 10.1016/j.parepi.2020.e00170, 32875128. 32875128 PMC7452100

[28] Nicholson M. J. and Butterworth M. H. , A Guide to Condition Scoring of Zebu Cattle, 1986, International Livestock Center (ILCA), https://books.google.com.et/books?hl=en%26lr=%26id=8rhIDD8XRBkC%26oi=fnd%26pg=PA11%26dq=Nicholson,+M.J.+and+Butterworth,+M.+H.+(1986).+A+Guide+to+Condition+Scoring+of+Zebu+Cattle.+International+Livestock+Center+(ILCA),+Addis+Ababa+Ethiopia.+ILRI+(aka+ILCA+and+ILRAD).&ots=f81lDCEg5S&sig=zKT8epPt7tvndMAiTL9oYtbm_GE&redir_esc=y#v=onepage&q&f=false.

[29] Saville W. J. and Wittum T. E. , Veterinary Epidemiology, Equine Internal Medicine, 2004, 2nd edition, John Wiley & Sons.

[30] OIE , Trypanosomosis, Manual of Diagnostic Tests and Vaccines for Terrestrial Animals (Terrestrial Manual), 2008, Office International des Epizooties, https://www.ojafr.com/main/attachments/article/127/OJAFR, 7(3) 51-57, 2017.pdf.

[31] Nevill E. M. , Tsetse Biology and Ecology: Their Role in the Epidemiology and Control of Trypanosomosis, S.G.A. Leak: Book Review, Journal of the South African Veterinary Association. (1999) 70, no. 4, 10.4102/jsava.v70i4.794.

[32] Lelisa K. and Meharenet B. , Anaemia Associated With Trypanosomes Infections in Cattle of West Gojjam Zone, Northwest Ethiopia, Veterinary Medicine International. (2021) 2021, no. 1, 5531537, 10.1155/2021/5531537, 34306606. 34306606 PMC8263224

[33] Paris J. , Murray M. , and McOdimba F. , A Comparative Evaluation of the Parasitological Techniques Currently Available for the Diagnosis of African Trypanosomiasis in Cattle, Acta Tropica. (1982) 39, no. 4, 307–316, 6131590. 6131590

[34] Griffin L. , An Improved Parasitological Technique for the Diagnosis of African Trypanosomiasis?, Transactions of the Royal Society of Tropical Medicine and Hygiene. (1978) 72, no. 2, 212–212, 10.1016/0035-9203(78)90072-X, 2-s2.0-0017795734. 653798

[35] Hosmer D. W. , Lemeshow S. , and Sturdivant R. X. , Logistic Regression Models for Multinomial and Ordinal Outcomes, Applied Logistic Regression, 2013, John Wiley & Sons, Inc, 269–311, 10.1002/9781118548387.ch8.

[36] Zeryehun T. and Abraham Z. , Prevalence of Bovine Trypanosomosis in Selected District of Arba Minch, SNNPR Southern Ethiopia, Global Veterinaria. (2012) 8, no. 2, 168–173.

[37] Efa D. A. , Bovine Trypanosomiasis Epidemiology and Tsetse Fly Density in Jimma Arjo District, East Wollega zone, Oromia Regional State, Ethiopia, Veterinary Medicine: Research and Reports. (2021) 12, 285–292, 10.2147/VMRR.S336585, 34745925. PMC8565986 34745925

[38] Adane M. , Teshome Y. , Jemberu W. T. , and Kebede N. , Epidemiology and Economic Impact of Bovine Trypanosomosis in Jawi District, Northwest Ethiopia, Scientific Reports. (2025) 15, no. 1, 1–14, 10.1038/s41598-025-90112-1, 40594968. 40594968 PMC12219873

[39] Robi D. T. and Diriba S. , Epidemiological Investigation of Bovine Trypanosomosis and Distribution of Vectors in Jimma zone, Ethiopia, Parasite Epidemiology and Control. (2021) 14, e00221, 10.1016/j.parepi.2021.e00221, 34430725. 34430725 PMC8367841

[40] Tulu D. , Gebeyehu S. , Aseffa N. , and Negera C. , Prevalence of Bovine Trypanosomosis and Associated Risk Factor in Jimma Horro District, Kellem Wollega Zone, Western Ethiopia, Journal of Veterinary Medicine and Animal Health. (2018) 10, no. 8, 185–191, 10.5897/JVMAH2018.0695.

[41] Tolawak D. , Berrie K. , and Pal M. , Prevalence of BovineTrypanosomosisand Associated Risk Factors in Jima Geneti District of the Horo Guduru Wollega Zone in Ethiopia, International Journal of Medical Parasitology and Epidemiology Sciences. (2022) 3, no. 1, 14–18, 10.34172/ijmpes.2022.04.

[42] Geiger A. , Ponton F. , and Simo G. , Adult Blood-Feeding Tsetse Flies, Trypanosomes, Microbiota and the Fluctuating Environment in Sub-Saharan Africa, International Society for Microbial Ecology Journal. (2015) 9, no. 7, 1496–1507, 10.1038/ismej.2014.236, 2-s2.0-84932196169, 25500509. PMC4478693 25500509

[43] Majekodunmi A. O. , Fajinmi A. , Dongkum C. , Picozzi K. , Thrusfield M. V. , and Welburn S. C. , A Longitudinal Survey of African Animal Trypanosomiasis in Domestic Cattle on the Jos Plateau, Nigeria: Prevalence, Distribution and Risk Factors, Parasites and Vectors. (2013) 6, no. 1, 10.1186/1756-3305-6-239, 2-s2.0-84882435744, 23958205. PMC3765779 23958205

[44] Hundessa N. , Esrael E. , Fesseha H. , and Mathewos M. , Study on Prevalence of Trypanosomosis in Cattle of Sodo Zuriya District, Wolaita Zone, Southern Ethiopia, Journal of Parasitology Research. (2021) 2021, no. 1, 4472480, 10.1155/2021/4472480, 34925912. 34925912 PMC8683203

[45] Efrem D. , Kassa T. , Kebede N. , and Worku T. , Seasonal Prevalence of Bovine Trypanosomosis and Trypanosome Species Distribution in Jimma Horo District, Oromia Regional State, Western Ethiopia, Parasite Epidemiology and Control. (2023) 20, e00280, 10.1016/j.parepi.2022.e00280, 36545242. 36545242 PMC9761842

[46] Degneh E. , Ashenafi H. , Kassa T. , Kebede N. , Shibeshi W. , Asres K. , and Terefe G. , Trypanocidal Drug Resistance: A Threat to Animal Health and Production in Gidami District of Kellem Wollega Zone, Oromia Regional State, Western Ethiopia, Preventive Veterinary Medicine. (2019) 168, 103–107, 10.1016/j.prevetmed.2019.03.017, 2-s2.0-85065160657, 31076189. 31076189

[47] Ushencho S. and Fekadu A. , Prevalence And Community Awareness Of Bovine Trypanosomiasis In Wolaita Zone Kindo Koysha Woreda, Southern Ethiopia, East African Journal of Biophysical and Computational Sciences. (2020) 1, no. 1, 23–34, 10.4314/eajbcs.v1i1.

[48] Abebe R. , Gute S. , and Simon I. , Bovine Trypanosomosis and Vector Density in Omo-Ghibe Tsetse Belt, South Ethiopia, Acta Tropica. (2017) 167, 79–85, 10.1016/j.actatropica.2016.12.016, 2-s2.0-85007107471, 28007483. 28007483

[49] Hill E. W. , O′Gorman G. M. , Agaba M. , Gibson J. P. , Hanotte O. , Kemp S. J. , Naessens J. , Coussens P. M. , and MacHugh D. E. , Understanding Bovine Trypanosomiasis and Trypanotolerance: The Promise of Functional Genomics, Veterinary Immunology and Immunopathology. (2005) 105, no. 3–4, 247–258, 10.1016/j.vetimm.2005.02.004, 2-s2.0-16244401956, 15808304. 15808304

[50] Degneh E. , A Cross-sectional Study of Bovine Trypanosomosis in Sayo District, Oromia Regional State, Western Ethiopia, International Journal of Nutrition and Food Sciences. (2018) 7, no. 2, 10.11648/j.ijnfs.20180702.13.

[51] Sheferaw D. , Abebe R. , Fekadu A. , Kassaye S. , Amenu K. , Data D. , Geresu E. , Olbamo G. , Anjulo A. , Yigebahal Z. , Estiphanos E. , and Mekuria S. , Prevalence of Bovine Trypanosomosis and Vector Density in a Dry Season in Gamo-Gofa and Dawuro Zones, Southern Ethiopia, Veterinary Parasitology: Regional Studies and Reports. (2019) 18, 100343, 10.1016/j.vprsr.2019.100343, 31796171. 31796171

[52] Gebeyehu S. and Robi D. T. , Epidemiological Investigation of Trypanosomosis in Livestock and Distribution of Vector in Dabo Hana District, Southwest Oromia, Ethiopia, Parasite Epidemiology and Control. (2024) 27, e00396, 10.1016/j.parepi.2024.e00396, 39720310. 39720310 PMC11667175

[53] Morrison L. J. , Steketee P. C. , Tettey M. D. , and Matthews K. R. , Pathogenicity and Virulence of African Trypanosomes: From Laboratory Models to Clinically Relevant Hosts, Virulence. (2023) 14, no. 1, 10.1080/21505594.2022.2150445, 36419235, 2150445. 36419235 PMC9815240

[54] Tola N. , Wagari A. , Lemu G. H. , Kedir M. , Gebremeskel H. F. , and Kebede I. A. , Trypanosome Infection in Cattle and Associated Vectors in Etang District of Gambella, Ethiopia, Journal of Parasitology Research. (2024) 2024, no. 1, 5548718, 10.1155/2024/5548718, 39144638. 39144638 PMC11323987

[55] Alemu J. and Gudina E. , Prevalence of Bovine Trypanosomosis and Its Associated Risk Factors in Selected Woredas of Gambella Regional State, South West Ethiopia, Journal of Agricultural and Crop Research. (2018) 6, no. 5, 97–104, 10.13140/RG.2.2.23973.42727.

[56] Eshetu E. , Barata B. , and Butako B. , Journal of Parasitology and Vector Biology The Prevalence of Bovine Trypanosomosis and Associated Risk Factors in Mareka Woreda of Dawuro Zone, Southern Ethiopia, Journal of Parasitology and Vector Biology. (2017) 9, no. 5, 39–46, 10.5897/JPVB2016.0265.

[57] Rowlands G. J. , Mulatu W. , Authié E. , d′Ieteren G. D. M. , Leak S. G. A. , Nagda S. M. , and Peregrine A. S. , Epidemiology of Bovine Trypanosomiasis in the Ghibe Valley, Southwest Ethiopia. 2. Factors Associated With Variations in Trypanosome Prevalence, Incidence of New Infections and Prevalence of Recurrent Infections, Acta Tropica. (1993) 53, no. 2, 135–150, 10.1016/0001-706X(93)90025-7, 2-s2.0-0027241055, 8098899. 8098899

[58] Torr S. J. and Mangwiro T. N. C. , Interactions Between Cattle and Biting Flies: Effects on the Feeding Rate of Tsetse, Medical and Veterinary Entomology. (2000) 14, no. 4, 400–409, 10.1046/j.1365-2915.2000.00257.x, 2-s2.0-0033665319, 11129704. 11129704

[59] Gona Z. , Teshale A. , and Tilahun A. , Study on Prevalence of Bovine Trypanosomosis and Density of Its Vectors in Three Selected Districts of Wolaita Zone, Southern Ethiopia, Journal of Veterinary Medicine and Animal Health. (2016) 8, no. 9, 128–135, 10.5897/JVMAH2016.0468.

[60] Green C. H. , The Effects of Odours and Target Colour on Landing Responses of *Glossina morsitans morsitans* and *G. pallidipes* (Diptera: Glossinidae), Bulletin of Entomological Research. (1993) 83, no. 4, 553–562, 10.1017/S0007485300039985, 2-s2.0-0027331644.

[61] Seyoum W. , Tora E. , Kore K. , and Lejebo F. , Seasonal Patterns: Bovine Trypanosomosis, *Glossina pallidipes* Density, and Infection in Rift Valleys of Gamo Zone, Southern Ethiopia, Frontiers in Veterinary Science. (2022) 9, 805564, 10.3389/fvets.2022.805564, 35359685. 35359685 PMC8961361

[62] Silva R. A. M. S. , Ramirez L. , Souza S. S. , Ortiz A. G. , Pereira S. R. , and Dávila A. M. R. , Hematology of Natural Bovine Trypanosomosis in the Brazilian Pantanal and Bolivian Wetlands, Veterinary Parasitology. (1999) 85, no. 1, 87–93, 10.1016/S0304-4017(99)00081-3, 2-s2.0-0033035910, 10447196. 10447196

[63] Van Den Bossche P. , Shumba W. , and Makhambera P. , The Distribution and Epidemiology of Bovine Trypanosomosis in Malawi, Veterinary Parasitology. (2000) 88, no. 3–4, 163–176, 10.1016/S0304-4017(99)00222-8, 2-s2.0-0033957095, 10714455. 10714455

[64] Stijlemans B. , De Baetselier P. , Magez S. , Van Ginderachter J. A. , and De Trez C. , African Trypanosomiasis-Associated Anemia: The Contribution of the Interplay Between Parasites and the Mononuclear Phagocyte System, Frontiers in Immunology. (2018) 9, 10.3389/fimmu.2018.00218, 2-s2.0-85042084628, 29497418. PMC5818406 29497418

[65] Bezabih M. , Shabula Z. , and Beyene N. , Prevalence of Bovine Trypanosomiasis in Dara District Sidama Zone, Southern Ethiopia, Journal of Parasitology and Vector Biology. (2017) 9, no. 9, 132–136, 10.5897/JPVB2015.0226.

[66] Biyazen H. , Duguma R. , and Asaye M. , Trypanosomosis, Its Risk Factors, and Anaemia in Cattle Population of Dale Wabera District of Kellem Wollega Zone, Western Ethiopia, Journal of Veterinary Medicine. (2014) 2014, no. 1, 6, 10.1155/2014/374191, 26464928. PMC4590855 26464928

[67] Mathewos M. , Endale H. , and Fesseha H. , Study on the Prevalence and Associated Risk Factors of Bovine Trypanosomiasis in Zaba Gazo Woreda, Southern Ethiopia, Research in Veterinary Science. (2022) 152, 53–57, 10.1016/j.rvsc.2022.07.009, 35926275. 35926275

[68] Santer R. D. , Developing Photoreceptor-Based Models of Visual Attraction in Riverine Tsetse, for Use in the Engineering of More-Attractive Polyester Fabrics for Control Devices, PLoS Neglected Tropical Diseases. (2017) 11, no. 3, e0005448, 10.1371/journal.pntd.0005448, 2-s2.0-85016958406, 28306721. 28306721 PMC5371378

[69] Santer R. D. , Vale G. A. , Tsikire D. , and Torr S. J. , Optimising Targets for Tsetse Control: Taking a Fly′s-Eye-View to Improve the Colour of Synthetic Fabrics, PLoS Neglected Tropical Diseases. (2019) 13, no. 12, e0007905, 10.1371/journal.pntd.0007905, 31830039. 31830039 PMC6907749

